# Cinderella Biodiversity: Three Decades of Monitoring Reveal Earlier Emergence and Increasing Abundance in Undervalued and Sparsely Recorded Flying Insects and Ballooning Spiders

**DOI:** 10.1111/gcb.71137

**Published:** 2026-10-06

**Authors:** Yoann Bourhis, Alice E. Milne, Chris R. Shortall, Matt Hampson, Dan Blumgart, William E. Kunin, James R. Bell

**Affiliations:** ^1^ Rothamsted Research Harpenden UK; ^2^ Rothamsted Insect Survey Rothamsted Research Harpenden UK; ^3^ Record LERC Suite 5 Parade Enterprise Centre Chester UK; ^4^ School of Biology University of Leeds Leeds UK; ^5^ School of Life Sciences Keele University Keele UK

**Keywords:** flying insects, insect decline, larval habitat, random forest, spiders, trophic guilds

## Abstract

Arthropod declines have been reported widely, and yet the spotlight has fallen on relatively large, colourful insects, rarely considering the abundant, small‐sized, yet charismatic flying insects and ballooning spiders we class as neglected and undervalued ‘Cinderella species’. This diverse set of arthropods contributes important ecosystem services, such as decomposition, pollination, pest regulation and nutrient cycling. We sampled a 30 year long archived arthropod collection, preserved in ethanol from the daily 12.2 m suction‐trap catches at four sites in the UK, to provide new data on more than 600,000 previously unidentified insects and spiders. Group or family‐level trends for the Coleoptera (beetles), Diptera (true flies), Hemiptera (true bugs) and Hymenoptera (bees, ants and wasps) as well as trends for seemingly less well‐known orders including the Psocoptera (barkflies), Dermaptera (earwigs) and Araneae (spiders) are provided. Our aim is to produce 30 year explainable trends in abundance and phenology for 71 Cinderella arthropod groups, and explore their functional trophic guild and habitat use during the immature stage. We trained random forest models to predict log abundance and phenology (Day of 5% catch) using a set of landscape, topography, weather‐related drivers. We show that the majority of arthropod taxa, as well as most trophic and habitat‐use groups, report either stable or increasing abundances, and stable or earlier phenologies. We also found that across taxa, earlier phenologies (54%–70% of taxa) and more abundant catches (55%–77%) are significantly related. These trends are mostly explained by temperature‐related drivers and secondarily by landcover, of which the strongest signal is improved grassland. Our study establishes a quantitative baseline for an undervalued, forgotten and often sparsely recorded segment of Britain's biodiversity.

## Graphical abstract

We provide 30 year trends in abundance and phenology for the less well known charismatic flying insects and ballooning spiders that we class as neglected and undervalued ‘Cinderella species’. We show that of the 71 groups studied, the majority of taxa, all trophic groups and nearly all habitat use groups report increasing abundances and stable or earlier phenologies. Across taxa, earlier phenologies tended to increase abundances. We also determine the roles of 33 scaled drivers (weather, landscape and year) in shaping Cinderella species trends, highlighting notable roles for temperature and the proportion of improved grassland. When using the image of a ballooning Erigone please cite Rothamsted Research.

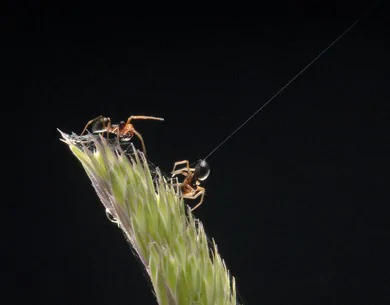

## 1 Introduction

Insects were among the first animals to colonize and establish niches in both terrestrial and freshwater ecosystems with recent evidence suggesting an Early Ordovician origin (Misof et al. 2014). Over the last 480 million years, insects have become the dominant life on Earth, reaching an unrivalled level of animal diversity that exceeds one million described species (Misof et al. 2014; Stork et al. 2015). Globally, the biomass of insects and other terrestrial arthropods exceeds the biomass of wild mammals by an order of magnitude (Rosenberg et al. 2023). Yet, with very few exceptions, insects have not received detailed attention beyond their taxonomic description, contrasting starkly with vertebrates (Cardoso et al. 2011; Leather 2013; Troudet et al. 2017). For example, nearly 100% of all bird species (11,195 sp.) have been assessed for IUCN's red listing, whilst only 0.01% of insects (13,442 sp.) have received the same treatment (IUCN 2025).

We define Cinderella insects as striking, intrinsically interesting and important yet neglected and undervalued despite their diversity, abundance and value to ecosystems and humanity. The term was first formally used for mammal conservation (Smith et al. 2012), but we extend that to have a broader meaning for species that are similarly charismatic when viewed under the microscope. The Coleoptera (beetles) are the most species‐rich order on Earth, accounting for 25% of all animal species (Stork et al. 2015). Beetles are a good benchmark to apply the ‘Cinderella species concept’ as they are vastly under‐recorded (Troudet et al. 2017). Apart from a few exceptions (Baur et al. 2020; Benton et al. 2002; Brooks et al. 2012; Gardiner and Didham 2020; Gebert et al. 2024; Hallmann et al. 2018, 2020; Harris et al. 2019; Homburg et al. 2019; Kotze and O'Hara 2003; van Noordwijk et al. 2017; Roth et al. 2020; Weiss et al. 2024) beetle families or species‐level population trends are almost unknown, despite the group's valuable ecosystem services that include decomposition, pollination, pest regulation, food and cultural services (Brooks et al. 2012; García‐Atencia et al. 2024; van Huis 2021; Morimoto 2020). The world's most speciose family, the rove beetles (Staphylindae), outnumber birds by a 6:1 species ratio (63,657 vs. 11,195), and yet there is an almost complete absence of long‐term abundance monitoring and species level trends (Betz et al. 2018; Boháč 1999) bar one notable exception (Ewald et al. 2024).

The Cinderella species concept could be imposed across most insect orders and families with little disagreement (Cardoso et al. 2011; Leather 2013; Troudet et al. 2017; Wang et al. 2021), although the butterflies, moths (Lepidoptera) and bees (Apoidea) are comparatively actively researched, featuring well in the Global Biodiversity Information Facility (GBIF) (Dorey et al. 2023; Garcia‐Rosello et al. 2023; Pinkert et al. 2022). These and other less well‐known insect orders largely depend on citizen science data to provide an assessment of their population status. Whilst citizen science data are vital and of unquestionable importance, they are unstructured or at best semi‐structured, usually recording presence‐only data (i.e., occupancy). As such, these data have inherent but not insurmountable challenges for statistical models to account for spatial, temporal, taxonomic and recorder biases, including absences (Pocock et al. 2017; Johnston et al. 2023; Bourhis et al. 2023, 2025; Powney et al. 2025).

Standardized, systematic long‐term entomological surveys that provide species level abundances are rare, with just a handful of global datasets available for research (Didham, Barbero, et al. 2020). For these types of surveys, ‘machines’ can reduce the spatial, temporal, taxonomic and recorder biases if they work passively, at a constant rate in a fixed location for a long period of time, ensuring standardization and a systematic approach (Bell et al. 2015, 2020). The Rothamsted Insect Survey (RIS) provides a unique 12.2 m height suction‐trap infrastructure that monitors the aerial insect fauna across a network of sites in Great Britain (Bell et al. 2015; Shortall et al. 2009). By biomass, the predominant group of insects caught are flies (Diptera) (Shortall et al. 2009; Grabener et al. 2020), though the largest group by number is aphids, which easily outnumber them (Bell et al. 2020).

The RIS suction‐trap network and its world‐class dataset was established in 1964 to monitor aphid migration, but in doing so generated a vast amount of other flying insect and spider samples, known as the ‘bycatch,’ which has been archived since 1974 (RIS 2025). This resource, estimated to include 150 million insect samples, provides an unprecedented opportunity to assess the impacts of land‐use and climate change. Population change and/or phenology estimates have been published for aphids, braconid parasitoids, overall fly abundance, ladybirds, wasps and general insect biomasses (Bell et al. 2015, 2020, 2011; Grabener et al. 2020; Lester et al. 2017; Martay et al. 2017; Pérez‐Rodríguez et al. 2015; Shortall et al. 2009, 2024; Stemkovski et al. 2023; Thackeray et al. 2010, 2016). However, there remains scope for examining a broader selection of species at higher resolutions, and thus it is essential that scientists report on changes in taxa that have yet to receive attention from the ~200,000 archived samples (Shortall et al. 2009, 2024; Petsopoulos et al. 2024).

In this paper we dramatically increase the coverage of the analysis of the RIS data to the furthest taxonomic level practicable, giving a more representative sample of the UK's flying insect fauna. We provide an analysis based on a sampling effort that resulted in the identification of more than half a million individuals across 71 insect families/superfamilies primarily drawn from the Diptera, Coleoptera, Hemiptera and Hymenoptera. For each of those 71 groups, we trained models to predict yearly abundances and phenology from environmental drivers. In doing so, we produce 31 year trends (1990–2020) in abundance and phenology providing a unique insight into the trends of underrepresented insects, including those of economic importance (e.g., gall midges (Cecidomyiidae), fungus flies (Sciaridae) and drain flies (Psychodidae) and weevils (Curculionidae)). We also include spiders (Araneae) and thus hereafter refer to arthropods.

We formulate a small number of expectations to anchor our analysis. H1: Given the well‐documented advancement of insect phenology with warming temperatures (Thackeray et al. 2010, 2016), we expect phenological trends to be broadly advancing across taxa. H2: Following the voltinism‐extension hypothesis (Macgregor et al. 2019), we expect taxa showing earlier phenologies to tend toward greater annual abundances. H3: Given the known thermal dependence of insect flight activity, we expect temperature‐related drivers to dominate our model explanations. H4: Given the widespread negative effects of agricultural intensification on insect communities (Seibold et al. 2019; Ewald et al. 2024), we expect agriculturally intensive land cover to negatively affect abundance. Beyond these hypotheses, the analysis is largely exploratory: Characterising abundance and phenological trends for 71 largely unstudied arthropod groups, and identifying the environmental drivers shaping them, is the primary contribution of this work.

## 2 Material and Methods

### 2.1 Field Sampling Methods

Suction traps measure the aerial abundance of small to medium‐sized flying insects. Set at a height of 12.2 m, these traps have been run by the Rothamsted Insect Survey (RIS), as part of a wider suction trap network, since 1964 (Bell et al. 2020; Shortall et al. 2009). Suction traps offer the most precise and standardized measurement of insect densities compared to other aerial capture techniques, consistently performing well across various meteorological conditions (Macaulay et al. 1988). To ensure accuracy, each trap in the network is annually calibrated to sample a standard volume of air (45 m^3^ min^−1^). As such, the networks represent the most comprehensive standardized long‐term data on insects in the World. However, only aphids have been consistently identified to species level over the last 60 years, leaving a large sample of undetermined insects of which we exploit.

During each year, traps provide a daily entomological sample during the main insect season (April to November) and weekly samples for the rest of the year (Bell et al. 2020; Shortall et al. 2009). The RIS maintains a network of traps in England (*n* = 12), of which four sites provided data for this study (Figure 1; see Table S1a): Newcastle (55.213; −1.685. Started 1965), Hereford (52.124; −2.638. Started 1971), Rothamsted (51.807; −0.360. Started 1964), and Starcross (50.630; −3.455. Started 1970). These sites were chosen because they were spatially separated across the region, providing a more comprehensive understanding of landscape change at different latitudes. Our sampling time series ran from 1990 to 2018, spanning 29 years, which is the largest resampling of the Rothamsted Archive, that began storing samples in 1974, ever undertaken. All archived samples are stored in an ethanol: Glycerol solution.

**FIGURE 1:**
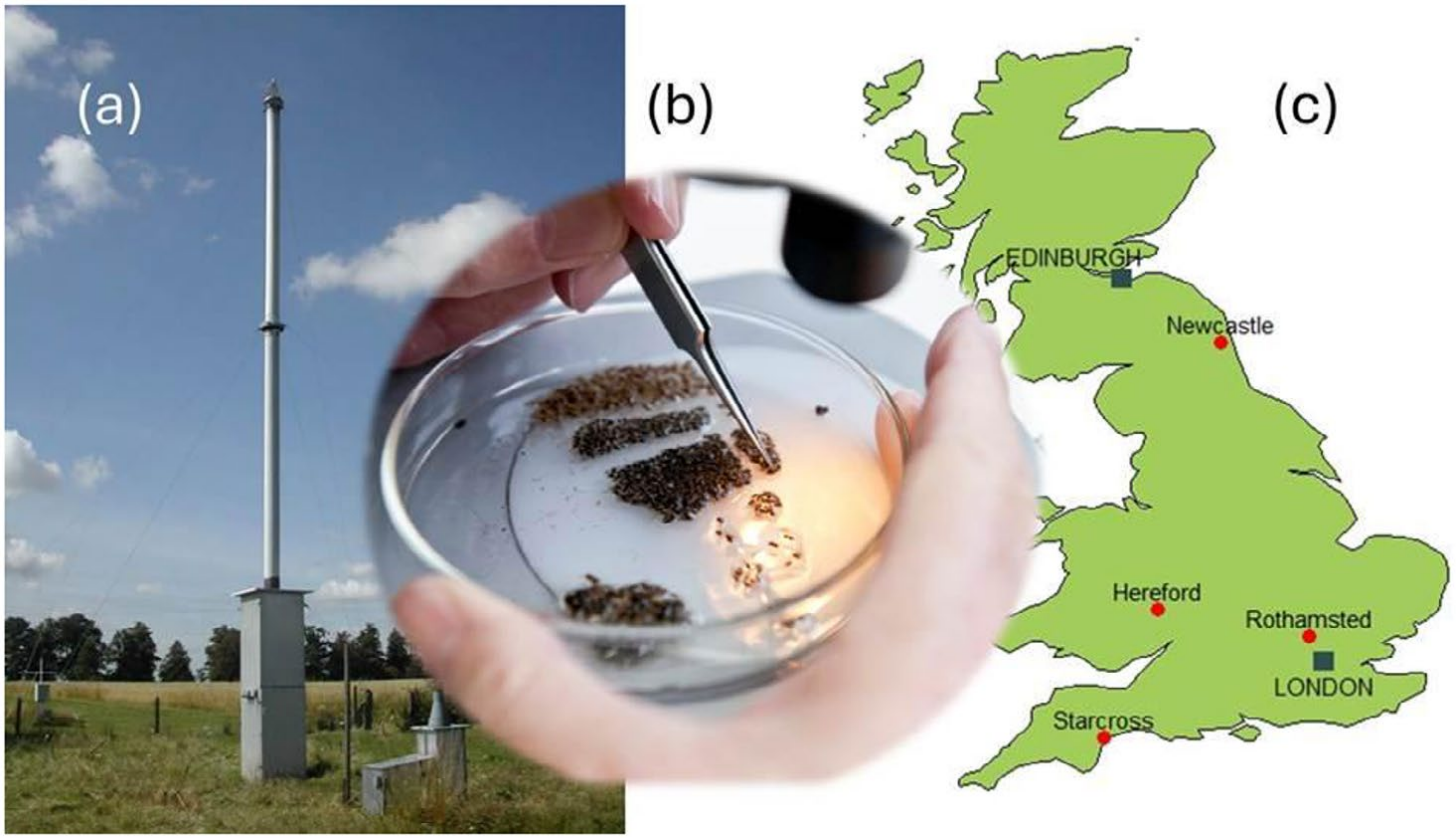
The 12.2 m suction trap (a) a typical Petri dish sample (b) and a map of the four sites studied (c) Map lines delineate study areas and do not necessarily depict accepted national boundaries.

### 2.2 Laboratory Sampling Methods

Between 9 and 11 years per site were sampled, five of which were common to all sites (1994, 2010, 2014, 2016 and 2018) (see Table S1b). The uneven sampling was a function of multiple demands on these data sets within the DRUID project, that include validation of flying insect numbers estimated by radar (Mungee et al. 2025). In each year, samples were identified every fourth day from 1st April to 28th September 1990–2018 (Shortall et al. 2025).

Whilst every effort was made to identify and count all insects in each sample, very small Thysanoptera (thrips), Parasitica (except the Cynipoidea and Ichneumonoidea), and Ptiliidae (featherwing beetles) were too challenging to count and/or identify and are not included.

Identifications were made to family or superfamily level, representing the finest taxonomic resolution achievable at the scale of this study (> 600,000 individuals across 30 years) and consistent with standard practice in large‐scale entomological monitoring at similar taxonomic breadth (Hallmann et al. 2020; Shortall et al. 2009; Seibold et al. 2019). While species‐level identification would be preferable for biodiversity assessment, family‐level abundance represents an ecologically meaningful unit of analysis: families share broadly similar larval ecology, trophic guild, body size range and voltinism, making family‐level abundance and phenology tractable metrics that should show coherent responses to environmental gradients.

### 2.3 Metrics

Building on the high temporal resolution of the individual counts, we derived two annual metrics for each taxon that captured year‐to‐year changes in arthropod population dynamics: (i) abundance, defined as total annual catch of individuals per taxon group and (ii) phenology, defined as the Julian date at which 5% of the year's individuals are captured.

We then subdivided the catch into trophic groups reflecting the adult dietary guild of each taxon (i.e., decomposers, herbivores, predators, omnivores, palynivores/nectivores, haematophagivores/nectivores and non‐feeding adults) (see Table S1c). Palynivores/nectivores were grouped together since those insect families (e.g., Scraptiidae, Anthomyiidae) are flower‐visiting and consume nectar for energy and pollen for protein. Haematophagivores/nectivores are biting flies in the families Ceratopogonidae, Culicidae and Simuliidae in which nectar feeding is a common activity in males and females, but haematophagy is exclusively within the female domain (Downes 1958). Due to recent work suggesting contrasting dynamics for terrestrial and freshwater invertebrates (van Klink et al. 2020), we also grouped taxa by their dependency on aquatic habitats during the immature phase. Thus, we included a life cycle trait describing the extent to which each taxon group contains species with terrestrial versus aquatic larval/instar stage, which we term hereafter ‘larval habitat trait’. Based on Smith's (1989) classification, we characterized families according to whether the species had entirely terrestrial instars/larvae, entirely aquatic larvae, majority aquatic larvae or minority of aquatic larvae (see Table S1c).

### 2.4 Drivers

We derived a set of 40 weather, modelled water quality and topography drivers, and 11 landscape drivers for the period 1990–2020 (see Table S1d). Although the insect observation data ends in 2018, we produced predictions for up to 2020, covering 3 decades and using the most recent land‐cover data available. The weather drivers were sourced from the BIOCLIM19 dataset linked to the Had‐UK grid (Hollis et al. 2019). The landscape drivers were mostly compositional (e.g., proportions of land given to arable, broadleaf or coniferous woodland) and derived from the UKCEH land‐cover maps. Modelled water quality drivers were sourced from Bell et al. (2021). We characterised each driver for each year at a 1 km × 1 km resolution. Using linear inverse distance weighting, the drivers were averaged around traps with buffer distances of 3 km and 10 km for the landscape‐related drivers, and 10 km for the remaining drivers. The reason behind the 2 distance buffers is that, unlike for weather drivers, we expect that landscape drivers could have some long and short‐range effects on insects. To reduce collinearity, drivers were filtered using a 75% correlation threshold, retaining only one variable from each correlated pair, preferring empirical variables over modelled ones, as well as preferring drivers with smaller radii around the trap. Finally, sampling year was included as a predictor to explicitly account for residual temporal trends not captured by the environmental drivers. Without this, unexplained temporal variation could be redistributed onto correlated predictors, distorting their estimated importance in subsequent model explanations (Jibiki and Fukuda 2024). See (Figures A3 and A4) for the shift in predictive performance (slightly better performance with year), as well as onto which the importance of year is preferably redistributed when it is removed. The final set included 33 drivers for subsequent analysis, which were centred and reduced (to a mean of 0 and standard deviation of 1) before they were used in the model (see Table S1d). We then explored their relationships with annual arthropod trends in abundance and phenology.

### 2.5 Models

Our aim was to model trends in insects and spiders at the finest taxonomic resolution possible. These groups can sometimes be sparsely recorded and as a consequence have been ignored, hence their Cinderella status. We trained random forest models (Breiman 2001) to predict the yearly log transformed abundance for each taxon. In the same fashion, we also trained random forest models to predict the yearly phenology of each taxon, using Day of 5% catch, hereafter referred to as simply DO5. Both sets of models built their predictions on the 33 environmental drivers (see Data Availability Statement) and were trained with the R package ranger (Wright and Ziegler 2017). For each metric, models were trained at the finest taxonomic resolution; the majority were family or superfamily level, rarely order level. Model performance was first assessed with a cross‐validation procedure (3 folds, 10 replicates), assessing RMSE on samples reserved as test dataset.

We then explored the predicted trends in both abundance and phenology. Analyses were conducted at two levels: overall arthropod community trends (Figures 4 and 8), and variation in trends across taxa and sampling sites (Figure 5). For abundance trends shown in Figures 4 and 8, we normalized the predictions to a reference value of 100 at the start of each trap time series to improve rate of change comparisons between groups. Phenological change was instead presented on the *y* axis as Julian days. Using that approach, trends were generated for two response traits: adult dietary guilds and larval habitat traits (Figure 8). We then assessed whether the two metrics exhibited related patterns. To assess the relationship between the rate of change in abundance against the rate of change in phenology (Figure 6), we used standardized major axis (SMA) regression from the smatr 3 R library (Warton et al. 2012), as both variables are measured with error. The SMA regression slope is directly calculated from the Pearson correlation coefficient and the standard deviations of the two variables, *x* and *y*, measuring its precision, but not accuracy. To identify the main drivers underlying these trends, we applied SHapley Additive exPlanations (SHAP, Lundberg and Lee 2017), a widely used approach for interpreting machine learning models such as random forests or artificial neural networks (Bourhis et al. 2023, 2025). SHAP assigns a directional effect to each feature for a given prediction, which summarise as directional importance values at the model level, thereby providing insights into how model inputs influence outputs in an otherwise ‘black‐box’ framework. In particular, we investigated which drivers appear to be key across taxa‐level models and whether their effects are consistent across arthropods. Both trend predictions and the SHAP explanations result from an ensemble approach (bagging, for bootstrap aggregation, i.e., resampling with replacement of the input data (Breiman 1996), with 30 replicates) which was used to estimate epistemic (data) uncertainty around trends and key driver identifications.

## 3 Results

The bycatch samples, comprising 618,779 arthropods across 71 arthropod families/superfamilies/orders, hereafter referred to as ‘groups’, comprising Diptera (82%), Coleoptera (8%), Hymenoptera (4%), Araneae (3%), Psocoptera (1%) and Hemiptera (1%) with the other 1% belonging to the Lepidoptera, Neuroptera, Trichoptera, Dermaptera and Ephemeroptera.

### 3.1 Trends in Abundance and Phenology

Figure 2 summarises how the two family‐level annual metrics distribute from year to year across the four traps. Hereford and Newcastle show relatively stable abundances and phenologies, whilst Rothamsted and Starcross show significantly increasing abundances and earlier phenologies.

**FIGURE 2:**
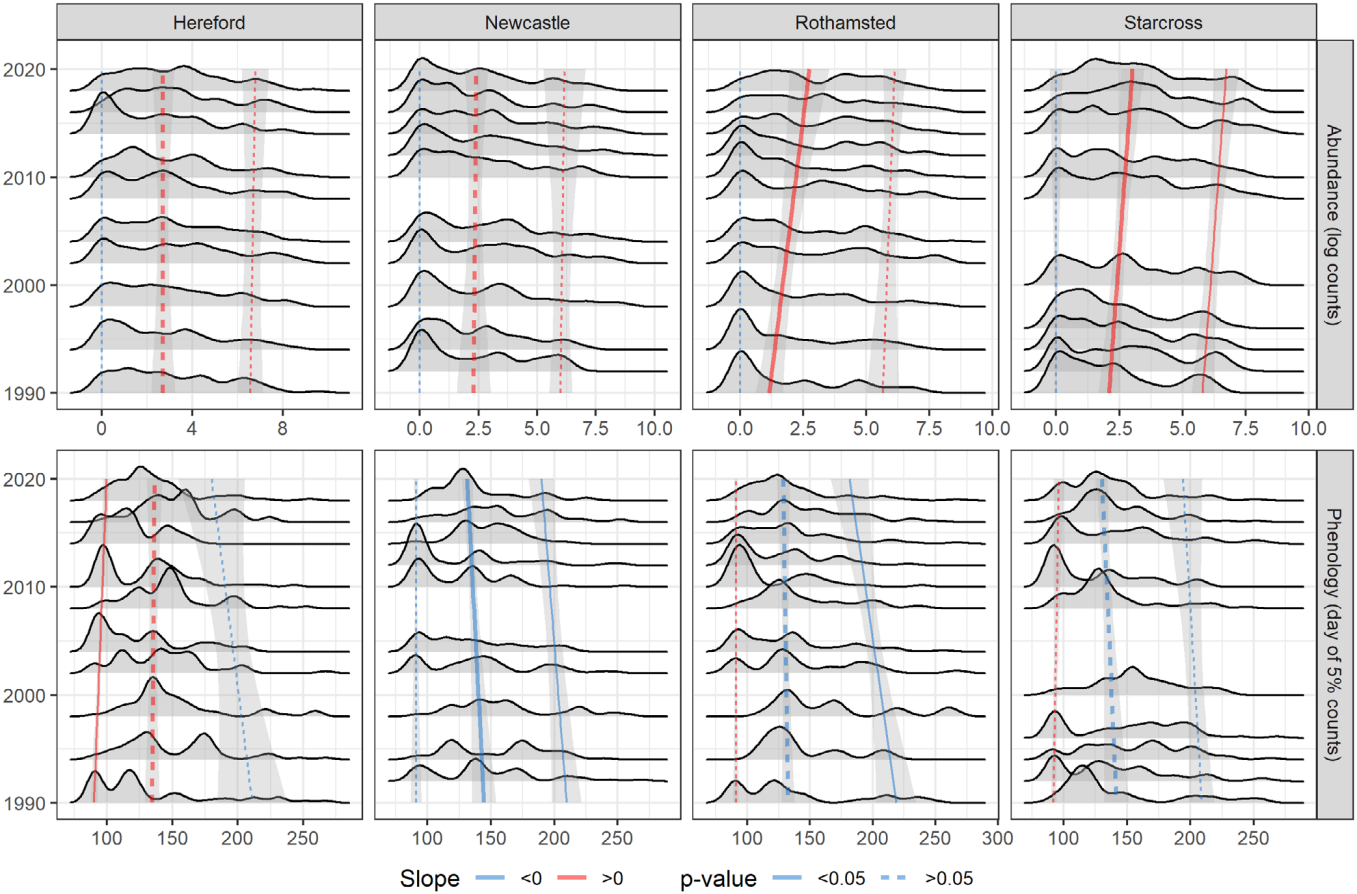
For each of the 71 groups annual abundance (log counts) and phenology (DO5) were derived at each of the four trapping sites. The figure shows the distributions of these metrics. The vertical lines show the quantile regressions of the 10th, 50th and 90th percentiles (from left to right respectively) with their 95% confidence interval, with solid lines marking significant (*p* < 0.05) slopes.

The random forest models exhibit contrasting predictive performances for the two metrics (Figure 3). While abundance is well predicted (RMSE = 0.93 log‐individual), the phenology metric is less accurately captured (RMSE = 31 days). We note a systematic regression‐to‐the‐mean bias in predicted vs. observed phenology (calibration slope < 1), with early DO5 predictions tending to be later than observed and late DO5 predictions that tend to be earlier than observed. This shrinkage indicates that the model does not capture the full variance present in the observed data. One candidate explanation is an artefact of the 4 days sampling interval, whereby the discrete detection window systematically smooths estimated emergence and decline dates toward the centre of the season. We tested this directly by subsampling to 8‐, 15‐, and 25‐days resolutions (Table A1). If the shrinkage were driven by sampling resolution, we would expect it to increase with coarser sampling; instead, the calibration slope and variance ratio remained essentially flat across resolutions (slope: 0.41 to 0.38; variance ratio: 0.465 to 0.448). The absence of a deterioration trend gives no basis to expect that finer‐than‐4 days sampling would reduce the shrinkage. We further tested this by linearly interpolating detections to an artificial daily sampling resolution, infilling days without catches, but the bias persisted (RMSE = 31 ± 0.2 days with or without interpolation). We also tested whether restricting weather covariates to the first half of the year (January–July)—the period plausibly most causally linked to early‐season phenology—improved early‐season predictions, but again found no meaningful change (RMSE = 31 ± 0.3 days with full‐year or half‐year weather data). Together, these results suggest that the observed shrinkage is not primarily a sampling‐resolution artefact, and more likely reflects limited predictive information in the available environmental covariates combined with unmodelled biological variability in emergence timing.

**FIGURE 3:**
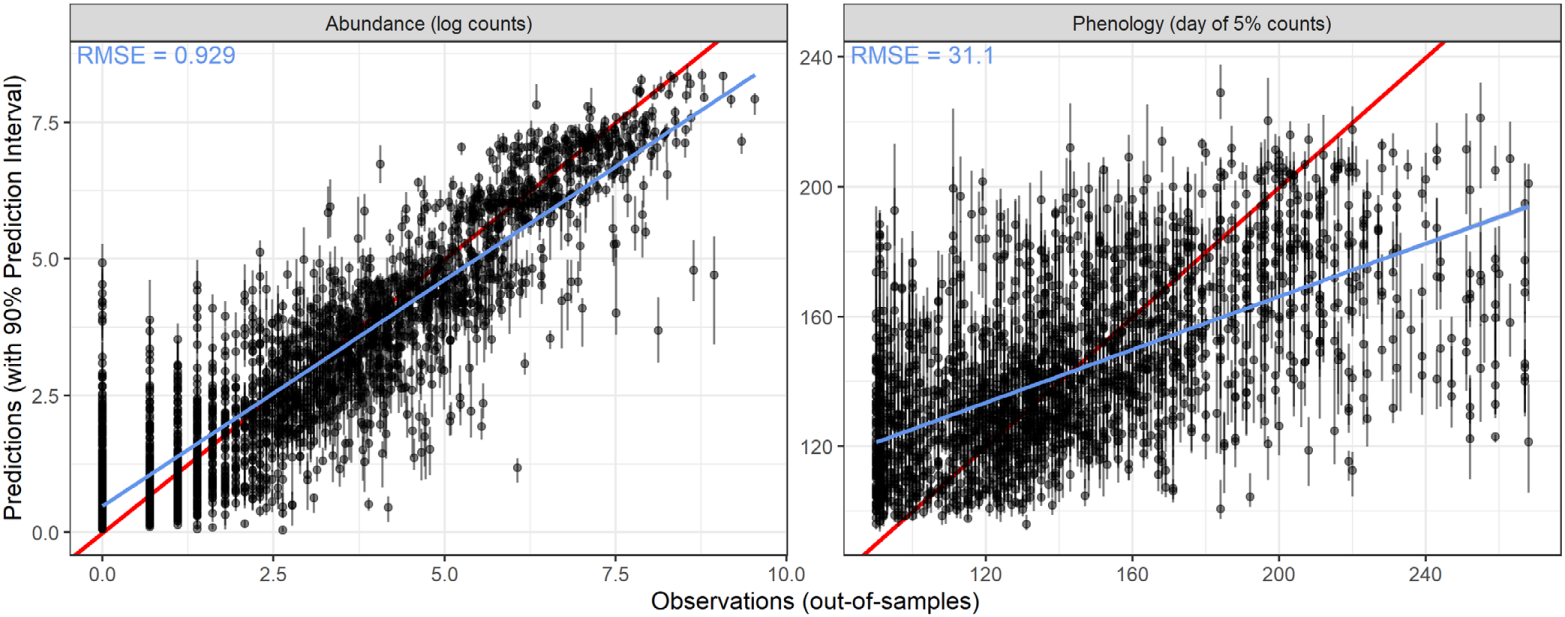
Predictions versus observations for the abundance (logged counts, left) and the phenology (DO5, right) using the test data sets (a quarter of the data unseen during training). Predictions are shown in their 50th percentile (dots), with error bars made of the 5th and 95th percentiles, with the root mean square error (RMSE) given in the top left of each panel. The red line is the identity line marking perfect predictions, while the blue line is the regression of predictions (50th percentile) against observations whose departure from the red line highlights the predictive bias.

Separately, we conducted two additional checks. First, we tested whether early‐ and late‐season phenologies were diverging over time by regressing each taxon's mean DO5 against its slope of change in DO5; no such pattern was evident (Figure A2). Second, excluding year from the predictor set left predictive performance and driver importance rankings effectively unchanged (Figures A3, A4), confirming that its inclusion as a nuisance control did not materially alter attribution to genuine environmental drivers.

Across all four traps, abundance increased significantly over the study period (+26%–+59%), while phenology advanced significantly at three of four traps (−1.4% to −3.6%; not significant at Hereford) (Figure 4). While these overall trends are significant, they obscure substantial diversity in taxon‐level responses (Figure 5). Some families and orders show marked declines in abundance across the four traps (e.g., Dolichopodidae), while others show delayed phenology (e.g., Pallopteridae). Nonetheless, most of the 71 taxonomic groups show local trends broadly consistent with the overall pattern of higher abundance and earlier phenology, albeit with considerable variation within and between groups.

**FIGURE 4:**
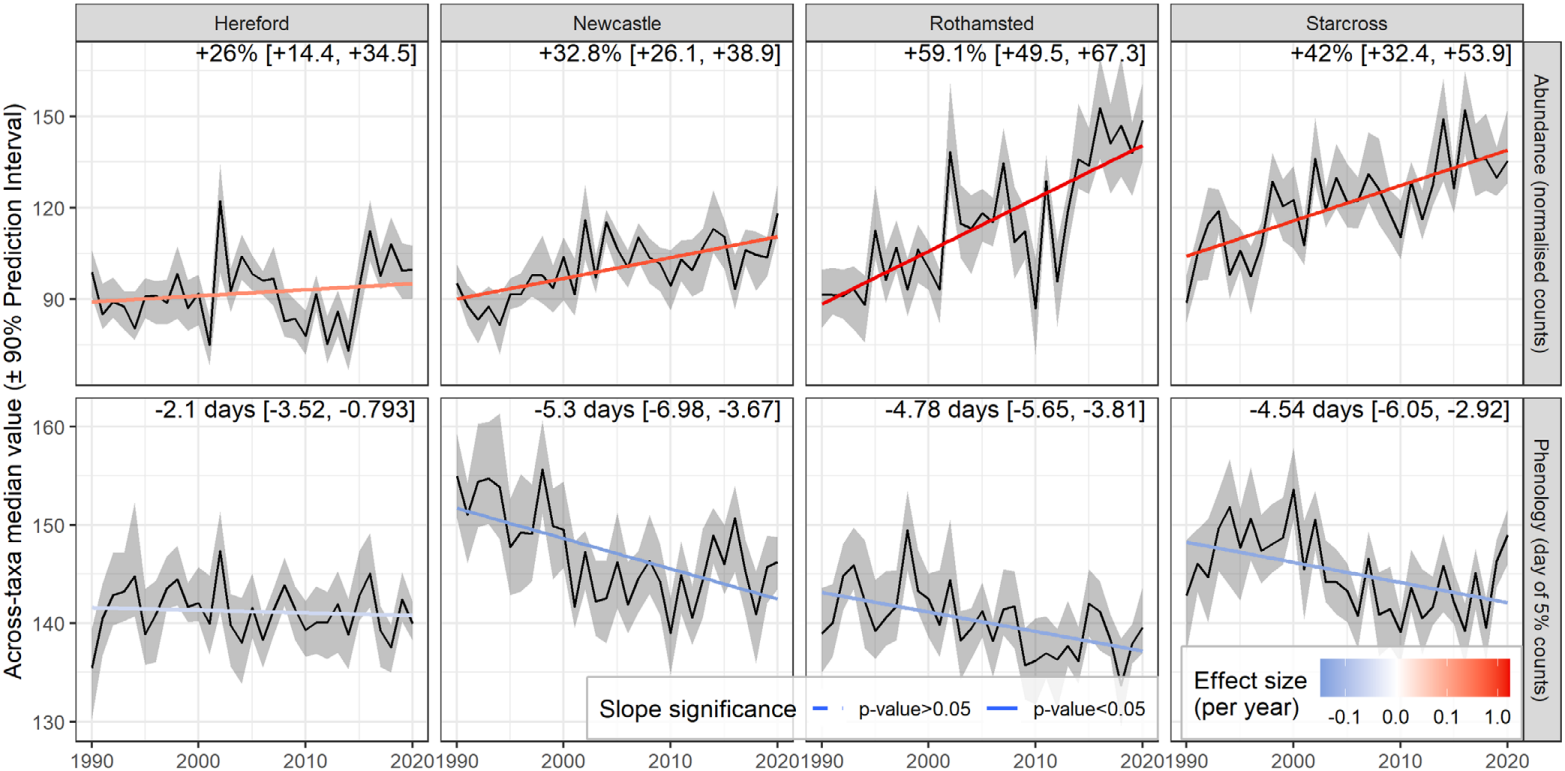
Predicted cross‐taxa average of normalized abundance and phenology (DO5) at the four trapping sites. Uncertainty is derived from the bootstrap replicates. Abundance refers to the number of arthropods per year, back‐transformed and normalized to 100 at the start of each time series. The phenology captures the Day of 5% arrival day without transformation. The cross‐taxa average change is shown in days in each panel, along with its 90% prediction interval (from *n* = 30 bootstraps) within brackets.

**FIGURE 5:**
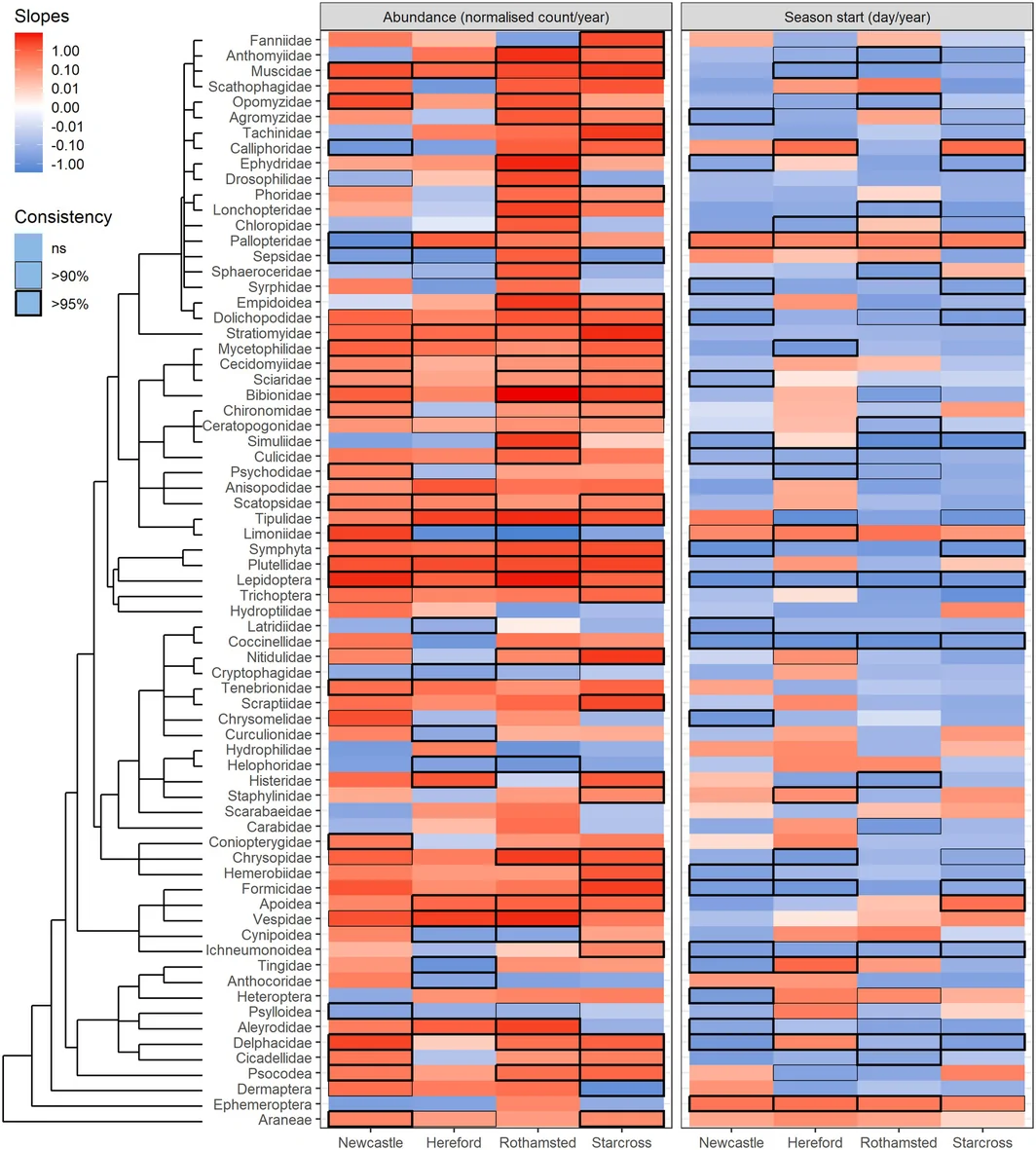
Summary of the taxa‐specific slopes derived from the normalised predicted abundance and DO5 phenology at the four trapping sites. The tiles outline highlight slopes across bootstrap replicates that are consistently positive (red); negative (blue). The taxonomic relationships are summarized by the tree on the left‐hand side. The (see Table S1c) provides the taxonomic hierarchies by order, family and guild with additional information on abundance.

### 3.2 Link Between Phenology and Abundance

At three of the four sites (Hereford, Newcastle, Rothamsted), the per‐taxon slope in phenology was significantly correlated with the per‐taxon slope in abundance (r = −0.27 to −0.32; Figure 6): taxa with more strongly advancing phenology tended to show greater increases in abundance, and a majority of taxa at these sites showed both earlier phenology (56%–68%) and increasing abundance (55%–77%). At Starcross, no significant correlation was detected (r = −0.11, 95% CI crossing zero), despite comparable individual proportions of taxa showing earlier phenology (66%) and increasing abundance (74%), indicating that phenological and abundance changes, while each common, were not linked at the taxon level at this site.

**FIGURE 6:**
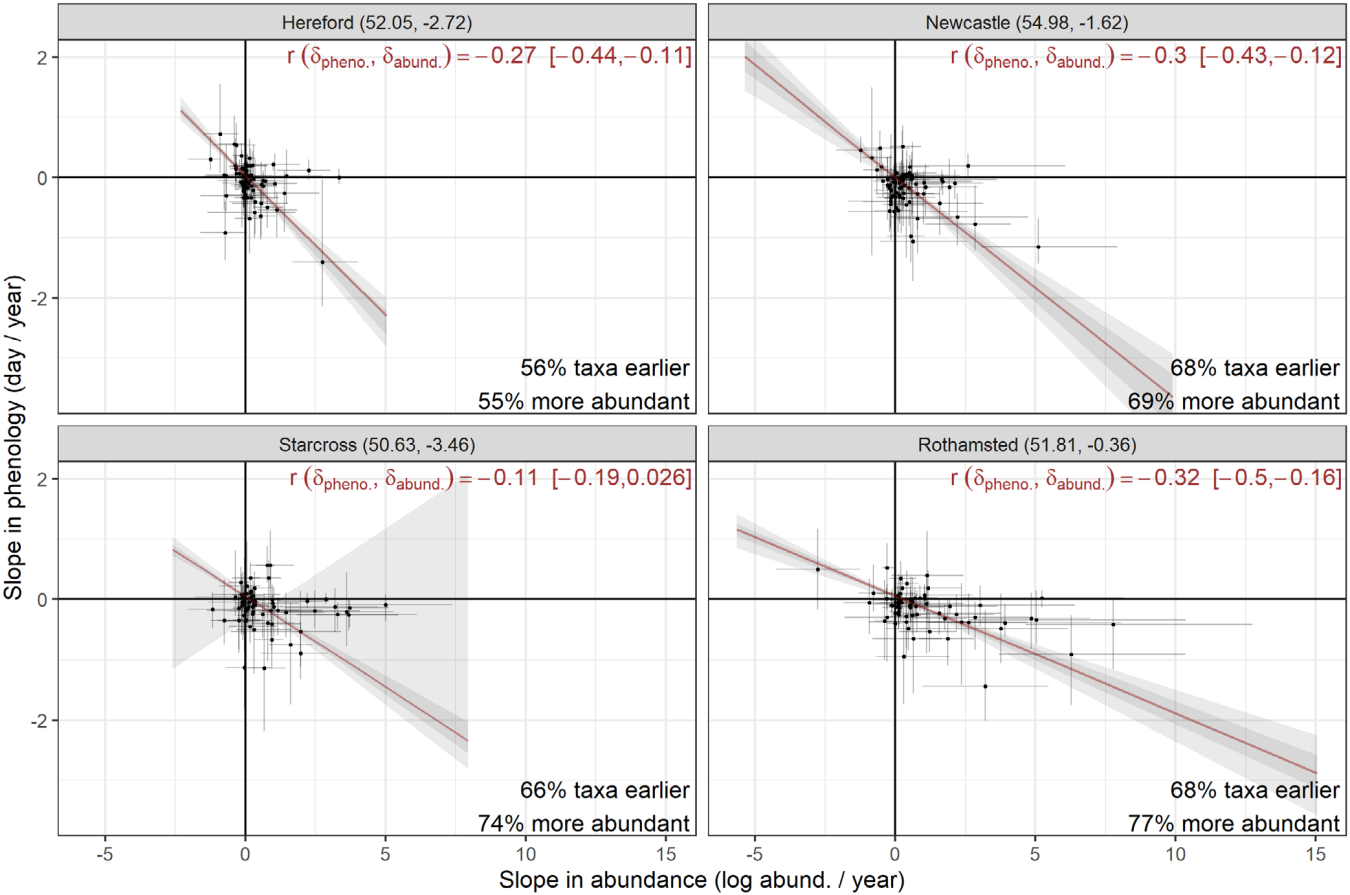
Results from the standardized major axis regression, showing distribution of slopes in abundance and phenology across all 71 taxa (the dots mark average slope across bags, while the segments show the 90% prediction interval). The red lines indicate the relationship between phenology and abundance with the shaded envelopes marking the 50% and 90% prediction intervals. The Pearson's *r* coefficient indicates the correlation with 90% CI from the bagged runs. If the CIs exclude zero from their range, then the correlation is significant for all but Starcross. The proportion of taxa showing greater numbers and earlier appearance is reported in the bottom‐right quadrant.

### 3.3 Explaining Trends: Drivers

Key drivers (see Table S1d) of arthropod log abundance and DO5 were determined by SHAP (Bourhis et al. 2025), which derives a directional effect of each driver on the model's response (Figure 7). Drivers are ordered by consistency of response across taxa. All drivers show a distribution of taxon‐level effects that are not exclusively positive or negative, instead acting differently across the 71 arthropod groups (i.e., with broad distributions of effects). Most drivers have distribution modes centred on zero, indicating no consistent community‐wide effect. However, a handful of drivers on the left‐hand side of Figure 7 have modal values that depart substantially from zero, indicating a largely consistent response across taxa. These are temperature‐related drivers characterising different aspects of thermal conditions throughout the year: within‐day variability (diurnal temperature range), seasonal extremity (temperature of the coldest quarter and frost days), and annual thermal consistency (isothermality and temperature seasonality). The proportion of improved grassland, grassland that has typically been managed to increased its performance for grazing stock, also shows a largely consistent effect, associated with earlier and more abundant flying arthropods, whereas previous‐year rainfall shows a largely consistent association with later phenology and lower abundance.

**FIGURE 7:**
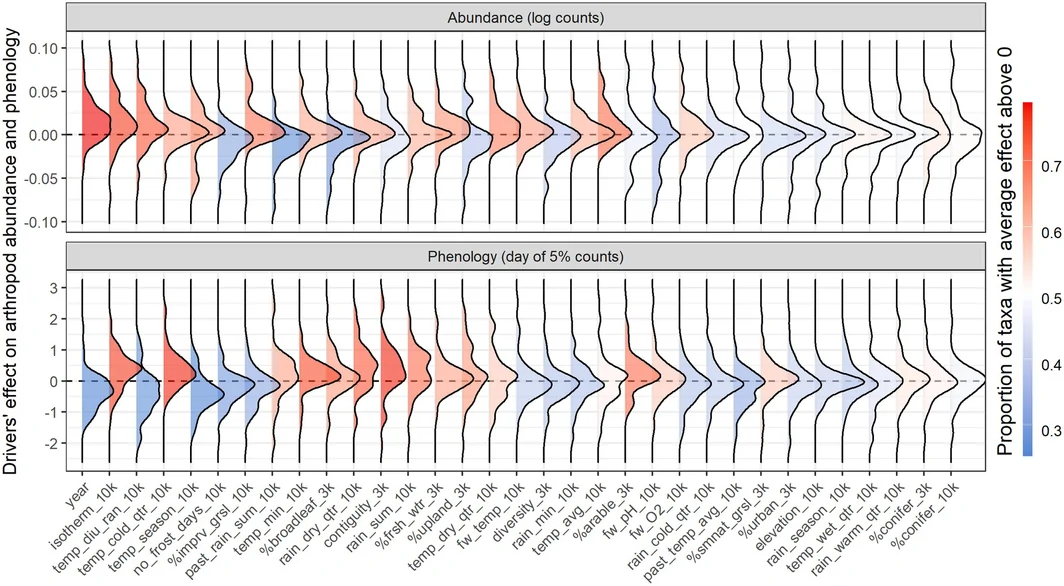
For each of the 71 taxa, the relationship between the 33 scaled drivers and their effects on log abundance (top) and phenology (DO5 in Julian days, bottom) contributed to a distribution of effects across all taxa. They are ordered from left to right according to the consistency of response, (i.e., the proportion of the distribution that excludes zero). The darker the colour, the more consistent the response. Narrow distributions indicate similar responses to a driver across taxa, while flatter distributions with longer tails represent drivers having varied effects on model responses.

### 3.4 Trends in Larval Habitats and Trophic Guilds

A largely consistent pattern of increased abundance was found across trophic guilds and larval habitat groups, with the notable exception of the decomposer group, which did not show any significant change (Figure 8). The trends in phenology are much more heterogeneous, however, with roughly half the groups showing no significant change (herbivores, non‐feeding adults, decomposers, minority aquatic larvae and mostly aquatic larvae) while the rest showed advancing phenologies (Figure 8). This does not contradict the taxon‐level phenology–abundance relationship reported in Section 3.2: that analysis identifies a relationship among individual taxa exhibiting directional phenological change, and null trends at the guild level are consistent with a mix of responsive and non‐responsive taxa being pooled within the same guild.

**FIGURE 8:**
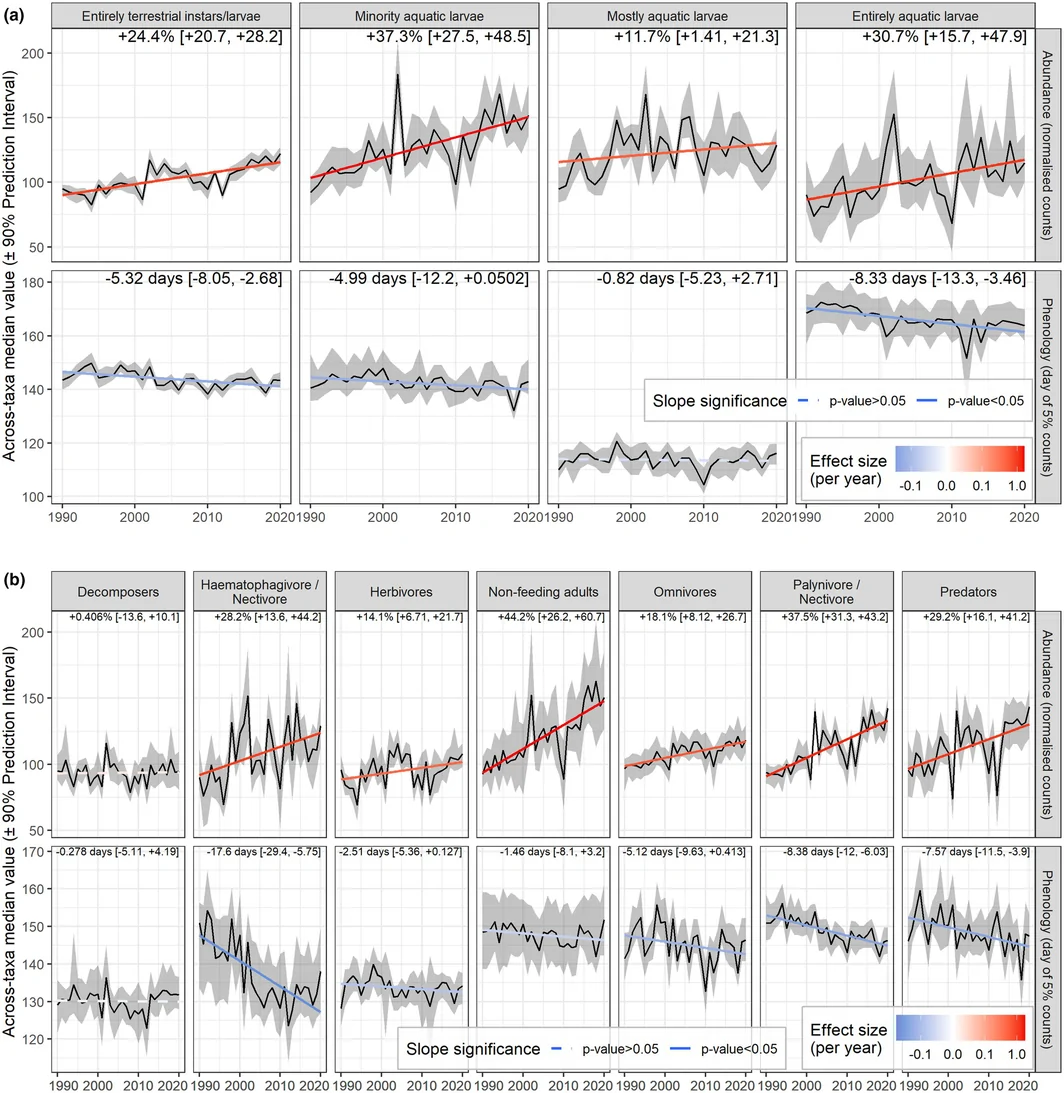
Trends in abundance and phenology (a) larval habitat (b) trophic guilds. Uncertainty is derived from the bootstrap replicates. The cross‐taxa average change is shown in days in each panel, along with its 90% prediction interval (from *n* = 30 bootstraps) within brackets.

## 4 Discussion

### 4.1 Main Findings

We analysed data on more than 600,000 arthropods from across four suction‐trap sites spanning 30 years. Our results show that abundance was stable for decomposers and increased for all other groups. The trends in phenology are more heterogenous, with many groups showing stable DO5 (Hereford (Figure 4); minority aquatic larvae and mostly aquatic larvae (Figure 8a); decomposers, herbivores, non‐feeding adults, omnivores (Figure 8b)), while the remaining groups (8 out of 15) show significantly earlier phenology. Earlier phenologies tended to be associated with a rise in the abundance of those taxa, exemplified by trends in Diptera (Agromyzidae, Culicidae, Dolichopodidae, Muscidae, Stratiomyidae), Hymenoptera (Formicidae, Symphyta), Coleoptera (Histeridae) and Hemiptera (Cicadellidae). However, this pattern was not a global phenomenon across all four sites and for all 71 groups studied. Notably, two of the most speciose insect families, the rove beetles (Staphylinidae) and weevils (Curculionidae), were much more mixed in their response.

Variation in temperature is a known dominant driver of both abundance and phenology; warmer temperatures drive earlier phenologies (Thackeray et al. 2010, 2016) and steadily rising isothermal currents—that are associated with warmer temperatures—drive increasing flying arthropod activity (Bell et al. 2013; Tielens and Kelly 2024; Mungee et al. 2025). In our study, temperature‐related drivers dominated the SHAP‐based attribution, with the five highest‐ranked environmental predictors all describing aspects of thermal variability (isothermality, temperature diurnal range, temperature of the coldest quarter, temperature seasonality and the number of frost days) followed by the proportion of improved grassland and previous‐year rainfall (Figure 7). Overall, our results suggest that variation in temperature is the dominant driver of both abundance and phenology, with agricultural landscape composition acting as a secondary influence.

### 4.2 Drivers of Abundance and Phenological Trends

Although most predictors exhibit substantial effects for at least some taxa, with their SHAP effect distributions being generally broad, their distributions are mostly centred near zero. Only a handful show consistent community‐wide effects, reflected by distribution modes departing from zero across the 71 groups. Interpretation of these drivers requires recognising that suction traps measure insects in flight rather than local ground‐level population size. The environmental drivers identified here should therefore be interpreted as influencing aerial activity, through changes in underlying abundance, dispersal behaviour, or both.

Temperature‐related variables account for the majority of these consistent community‐level effects. Five of the highest‐ranked drivers describe thermal conditions at different timescales: Within‐day variability (diurnal temperature range), seasonal extremity (temperature of the coldest quarter and frost days), and annual thermal consistency (isothermality and temperature seasonality). Together, these drivers are associated with higher aerial abundance and earlier phenology across most taxa, consistent with warming conditions extending the flight season (Bell et al. 2013; Harvey et al. 2020).

Among the non‐temperature drivers, the proportion of improved grassland stands out, perhaps because it is on a dramatic rise (Suggitt et al. 2023), and shows a positive association with aerial abundance and earlier phenology. This result is initially surprising and contrary to our expectation, as improved grassland is botanically impoverished and unfavourable for many insect groups compared to semi‐natural grassland (Ridding et al. 2025), although this does not always lead to uniform change in species composition or abundance (van Noordwijk et al. 2017; Beckmann et al. 2019; Powell et al. 2024; Shipley et al. 2024). Whilst recognising that other drivers may also be influencing levels of habitat fragmentation, a plausible explanation is that greater proportions of improved grassland increase habitat fragmentation, reducing local population sizes while promoting dispersal between habitat patches (Denno et al. 1996; Matthysen 2012; Cardoso et al. 2024). Such opposing effects on abundance and movement are well recognised in activity‐density measures, making higher trap catches as readily interpreted as increased flight activity or increased underlying population size. Previous‐year rainfall also emerges as an important driver, consistent with lagged effects of larval‐season conditions on adult aerial abundance in the following year (Bell et al. 2015), for example through increased plant productivity. Note that we lacked drivers for river typologies, particularly the geology of the riverbed, which have explained trends in abundance for aquatic macroinvertebrates (Powell et al. 2022). We also did not detect a high‐ranking impact of urban land‐covers, even though Great Britain is 8% urbanised (ONS 2018), despite previous studies showing profound impacts of urbanisation on insect abundance (Fenoglio et al. 2020; Piano et al. 2020).

### 4.3 Phenology‐Abundance Relationship

The association between earlier phenology and greater abundance, significant at three of the four sites, is consistent with the hypothesis that temperature‐driven advances in phenology yield more individuals. Extended warmer conditions allow multivoltine species to complete additional generations within a season, translating shifts in seasonal timing into population gains, whereas univoltine species may gain comparatively little from a longer season (Macgregor et al. 2019). This interpretation is not without caveats, not least because insects have an upper threshold for flight (Johnson 1969). The lost generation hypothesis (van Dyck et al. 2015) predicts that earlier emergence may instead increase mortality when deteriorating late‐season conditions prevent an additional generation from completing development. Bell et al. (2015) showed that advancing aphid phenologies do not necessarily result in higher annual abundance, even in highly multivoltine species. Nevertheless, across the broad range of taxa considered here, earlier phenology was generally associated with higher abundance, with the association reaching significance at three of the four study sites, indicating that this pattern is broadly, though not universally, consistent across locations.

### 4.4 Reconciling Divergent Abundance Trends

Our finding that most arthropod groups show stable or increasing abundance at 12.2 m contrasts with the widespread declines reported for ground‐level insect and spider communities (Hallmann et al. 2018, 2020; van Klink et al. 2020; Skarbek et al. 2021; Samu et al. 2023). We suggest that this apparent discrepancy reflects differences in the community components sampled and in the temporal baselines considered, making our findings complementary rather than contradictory to the existing evidence.

To understand the difference between near ground level surveys that generally report declines, and radar and suction trap data that generally report stable or increasing populations, we invoke the concept of ‘appetitive’ vs. ‘migration’ movements, to distinguish between vastly different flight modes (Johnson 1969). Below the flight boundary layer (FBL) ceiling where insects exert active control over their flight speed and direction, routine foraging flight and commuting take place, aka appetitive flight, typically between 1 and 10 m height (Taylor 1974). Here, most insect surveys are conducted either at ground level or a few meters above (e.g., malaise traps). Beyond the FBL ceiling and within the convective boundary layer, migratory flight takes place in which insects are subject to prevailing atmospheric conditions (Wainwright et al. 2017). These migratory events are far from routine, but exceptional in the life history of those insects. In this layer, radar and suction trap surveys are conducted. In short, we make the necessary distinction between sampling appetitive and migrants flying insects because migrants are in different pre‐programmed physiological and behavioural states that yield different community compositions than insects within the FBL, particularly those on the ground.

At 12.2 m, the traps primarily sample insects engaged in migration, a component of the community that differs from ground‐level assemblages in species composition and ecological function. Above the FBL, aerially‐active arthropods caught in a suction trap catch are an admixture of local to continental populations concentrated in a single catch. A species may therefore decline locally while maintaining or even increasing aerial activity, if habitat disturbance promotes dispersal (Denno et al. 1996; Matthysen 2012). Divergent results between the two approaches are therefore not only expected but ecologically informative. The aerial community is also dominated by mobile generalists, the very taxa reported to increase as communities homogenise under global change (van Klink et al. 2023; Seibold et al. 2019), meaning our divergent result may in fact be consistent with processes already identified in ground‐level studies. Rather than providing an alternative estimate of insect population trends, aerial monitoring captures a complementary dimension of community change; the abundance and movement of insects within a key dispersal layer.

Differences in temporal baselines probably also contribute to the discrepancy (Didham, Basset, et al. 2020). The most severe phase of UK agricultural intensification preceded our 1990 start date (Ridding et al. 2025; Suggitt et al. 2023), meaning our trends are measured relative to an already‐degraded baseline. Longer time series are rare, but Neff et al. (2026) have shown that mean occupancies and species richness trends over recent decades can be substantially more positive than over the full twentieth‐century trajectory. Had our time series begun several decades earlier, it would likely have captured the initial declines associated with rapid post‐war agricultural intensification, which encouraged an increase in domestic food production with a causal reduction in habitat diversity following the implementation of the Agriculture Act 1947 (Robinson and Sutherland 2002). As such, our results cannot be interpreted as evidence that aerial arthropod communities are in a favourable state of conservation.

### 4.5 Limitations

Spatial replication is limited to four sites. Although these span 5° of latitude and a broad environmental gradient across Great Britain, they cannot capture the full diversity of Britain's arthropod communities or landscapes. Likewise, family‐level identifications, while necessary at this scale and consistent with comparable monitoring studies (Hallmann et al. 2020; Seibold et al. 2019), cannot distinguish between species‐level declines and compositional turnover, with widespread generalists replacing habitat specialists. Our findings should therefore be interpreted primarily at the functional‐group level which link to vital ecosystem services (e.g., decomposition, pollination, pest regulation, nutrient cycling) rather than as evidence for species‐level conservation status.

Phenological estimates are substantially less precise than abundance estimates and exhibit systematic bias, with early DO5 values tending to be overestimated and late values underestimated. This bias is not explained by the 4 days sampling period, but more likely reflects limitations in the predictive information contained in the available environmental covariates, together with unmodelled biological variability. Importantly, the observed bias depends on the magnitude of DO5 rather than on sampling year (Figure A1). Although this limits the accuracy of absolute phenological estimates, it is unlikely to introduce systematic errors into temporal trend analyses.

## 5 Conclusions

Our study provides the broadest taxonomic assessment to date of aerial arthropod abundance and phenological trends from a standardised long‐term monitoring network. The Cinderella arthropods documented here, abundant, functionally important and largely ignored in most surveys, show broadly stable or increasing aerial abundance over the past three decades. Temperature emerged as the dominant community‐wide driver, while agricultural landscape composition exerted a secondary influence. Whether these patterns reflect genuine population increases, increased dispersal from fragmented habitats, or shifts in species composition remains unresolved and highlights the need to integrate aerial and ground‐based monitoring. Abundance and biodiversity represent distinct dimensions of ecological change. By documenting the former across an exceptionally broad range of aerial arthropods, our study establishes a quantitative baseline against which future trajectories of this understudied component of biodiversity can be assessed.

## Author Contributions

**Yoann Bourhis:** conceptualization, methodology, data curation, investigation, formal analysis, writing – review and editing. **Alice E. Milne:** conceptualization, data curation, methodology, writing – review and editing, supervision, project administration, funding acquisition. **Chris R. Shortall:** methodology, conceptualization, data curation, investigation, supervision. **Matt Hampson:** methodology, investigation, writing – review and editing. **Dan Blumgart:** methodology, investigation, writing – review and editing. **William E. Kunin:** conceptualization, writing – review and editing, project administration, funding acquisition. **James R. Bell:** conceptualization, methodology, investigation, writing – original draft, project administration, supervision, funding acquisition.

## Funding

This work was supported by Biotechnology and Biological Sciences Research Council (BBS/E/C/000J0200, BBS/E/RH/23NB0006). Natural Environment Research Council (NE/V006916/1, NE/V00686X/1).

## Conflicts of Interest

The authors declare no conflicts of interest.

## Supporting information


**Figure A1:** Prediction residuals shown per traps along a temporal axis. No obvious temporal or spatial trend appears here to explain the bias observed in the phenological model (Figure 3).
**Figure A2:** Slopes in response metric (log abundance and DO5) versus average response metric, predicted and observed, per taxa, trap and year. No obvious relationship appears here. The most abundant taxa are not preferentially getting more abundant, while the earliest taxa are not preferentially getting earlier.
**Figure A3:** Distribution of change in RMSE (per taxa) when year is removed from the predictors. More taxa are seeing better performances with year included (positive RMSE change).
**Figure A4:** Change in predictors' importance (absolute SHAP value) when year is removed from the predictors. All predictors become more important as year effect is distributed across the board. From top to bottom the predictors are ordered by average importance change (black dots) in the abundance models. Cross taxa variation is shown in thick blue as standard error of the mean, while the black lines show the SEM accounting for epistemic uncertainty (i.e., +average intra taxa variance).
**Table A1:** Performance of the phenology model after progressively reducing the temporal sampling resolution of the response variable. The models (random forest ensembles) were retrained on data subsampled to each sampling interval and evaluated against observations at the corresponding resolution. Although absolute RMSE decreased as the prediction target became simpler, normalised RMSE, calibration slope, and variance ratio changed only marginally, indicating that reduced sampling frequency contributes little to the observed regression‐to‐the‐mean bias.

**Table S1a:** Families and orders.
**Table S1b:** Sampled years by site.
**Table S1c:** Insect guilds and traits.
**Table S1d:** Environmental drivers.

## Data Availability

The data that supports the findings of this study is available from the Rothamsted Repository: Shortall et al. (2025): https://doi.org/10.23637/h60r48rn. The R code to train and run the Random Forest is available on gitlab and distributed under GNU GPL3 licence (https://gitlab.com/Yo‐B).
