# Supplementary material for: Cinderella Biodiversity: Three Decades of Monitoring Reveal Earlier Emergence and Increasing Abundance in Undervalued and Sparsely Recorded Flying Insects and Ballooning Spiders
Source: Glob Chang Biol. 2026 Oct 6;32(10):e71137. doi: 10.1111/gcb.71137 (PMC13643034; doi:10.1111/gcb.71137)
Supplement: Supplementary file 1 [file GCB-32-e71137-s002.docx]

Supporting Information

S1- Data

Please refer to document supp.xlsx for:

**Table S1a**: Families and orders

**Table S1b**: Sampled years by site

**Table S1c**: Insect guilds and traits

**Table S1d**: Environmental drivers

S2- Supplementary figures

**Figure A1**: Prediction residuals shown per traps along a temporal axis. No obvious temporal or spatial trend appears here to explain the bias observed in the phenological model (Figure 3).

**Figure A2**: Slopes in response metric (log abundance and DO5) versus average response metric, predicted and observed, per taxa, trap and year. No obvious relationship appears here. The most abundant taxa are not preferentially getting more abundant, while the earliest taxa are not preferentially getting earlier.

**Figure A3**: Distribution of change in RMSE (per taxa) when year is removed from the predictors. More taxa are seeing better performances with year included (positive RMSE change).

**Figure A4**: Change in predictors’ importance (absolute SHAP value) when year is removed from the predictors. All predictors become more important as year effect is distributed across the board. From top to bottom the predictors are ordered by average importance change (black dots) in the abundance models. Cross taxa variation is shown in thick blue as standard error of the mean, while the black lines show the SEM accounting for epistemic uncertainty (i.e. +average intra taxa variance).

**Table A1:** Performance of the phenology model after progressively reducing the temporal sampling resolution of the response variable. The models (random forest ensembles) were retrained on data subsampled to each sampling interval and evaluated against observations at the corresponding resolution. Although absolute RMSE decreased as the prediction target became simpler, normalised RMSE, calibration slope, and variance ratio changed only marginally, indicating that reduced sampling frequency contributes little to the observed regression-to-the-mean bias.

| **Sampling period (days)** | **RMSE (days)** | **Normalised RMSE** | **R²** | **Calibration slope** | **Variance ratio** |
| --- | --- | --- | --- | --- | --- |
| 4 | 31.7 | 0.804 | 0.361 | 0.409 | 0.465 |
| 8 | 30.9 | 0.803 | 0.362 | 0.411 | 0.466 |
| 15 | 29.3 | 0.807 | 0.357 | 0.408 | 0.466 |
| 25 | 26.8 | 0.826 | 0.328 | 0.383 | 0.448 |
